# Non-technical skills of Norwegian medical students at different training sites: a comparative, observational cohort study

**DOI:** 10.1186/s12909-024-05597-7

**Published:** 2024-06-04

**Authors:** Katrine Prydz, Peter Dieckmann, Hans Fagertun, David Musson, Torben Wisborg

**Affiliations:** 1 Department of Clinical Medicine, Interprofessional Rural Research Team, Faculty of Health Sciences, University of Tromsø– the, Arctic University of Norway, Hvalrossveien 12, Hammerfest, 9602 Norway; 2 Hammerfest Hospital, Finnmark Health Trust, Hammerfest, Norway; 3 Copenhagen Academy for Medical Education and Simulation (CAMES), Center for Human Resources and Education, Capital Region of Denmark, Denmark and University of Stavanger, Copenhagen, Norway; 4 Faculty of Health Sciences, Department of Quality and Health Technology, University of Stavanger, Stavanger, Norway; 5 Department of Public Health, Copenhagen University, Copenhagen, Denmark; 6 Capturo AS, Skjetten, Norway; 7 Department of Anesthesia, Faculty of Health Sciences, McMaster University, Hamilton, ON Canada

**Keywords:** Medical student, Non-technical skills, NorMS-NTS, Decentralized education, Rural education

## Abstract

**Purpose:**

Mastering non-technical skills (NTS) is a fundamental part of the training of new physicians to perform effectively and safely in the medical practice environment. Ideally, they learn these skills during medical school. Decentralized medical education is being implemented increasingly worldwide. Two of the three training sites studied, Bodø (a regional hospital) and Finnmark (a rural local hospital), implemented decentralized medical education. The third training site was the main campus in Tromsø, located at an urban university hospital. The training in Finnmark emphasised training in non-technical skills using simulation to a larger extent than the two other university campuses. This study aimed to compare the NTS performance of medical students in their last year of education at three different training sites of the same university.

**Methods:**

This blinded cohort study included students from the three training sites who participated in identical multi-professional simulations over a six-year period. Eight raters evaluated the video recordings of eight students from each training site using the Norwegian Medical Students Non-Technical Skills (NorMS-NTS) tool. The NorMS-NTS tool, which comprises four categories and 13 elements, assesses the NTS of Norwegian medical students and assigns an overall global score. Pairwise significant differences in the NTS performance levels between the training sites studied were assessed using Tukey’s test.

**Results:**

The overall NTS performance levels of the medical students from Finnmark (mean 4.5) were significantly higher than those of the students from Tromsø (mean 3.8) and Bodø (mean 3.5). Similarly, the NTS performance levels at category-level of the students in Finnmark were significantly higher than those of the students from Bodø and Tromsø. Except for one category, no significant differences were observed between the students from Bodø and Tromsø in terms of the overall or category-level NTS performance.

**Conclusion:**

The NTS performance levels of the medical students from Finnmark, which implements rural, decentralized medical education, were significantly higher than those of the students from Tromsø and Bodø.

**Supplementary Information:**

The online version contains supplementary material available at 10.1186/s12909-024-05597-7.

## Introduction

The first medical school in Northern Norway, established at UiT – the Arctic University of Norway (UiT) 50 years ago [1], was the first rural-oriented medical education model in Europe to recruit physicians to the underserved population of northern Norway with the intent to improve the health care standards in the region [1]. The university implemented one year of training in rural general practice and local hospitals outside the campus [1]. Moreover, the university also prioritized applicants from Northern Norway [1] owing to an expected Salmon Effect, which hypothesizes that physicians, similar to salmons, return to the region they grew up [2]. A previous study concluded that the medical education program in Tromsø facilitate the recruitment of physicians to the northern regions [1].

Later, students in Tromsø were able to take the last two years of their medical education program in other parts of Northern Norway; Bodø and Finnmark. These two years consist of both classroom and clinical teaching. In 2009 UiT developed *the Bodø model* [3], a decentralized model wherein 24 medical students from UiT completed the sixth and last year of undergraduate medical education in Bodø, in addition to the placement in the fifth year of study [4]. The Bodø model aimed to address the limitations of clinical training capacity available in Tromsø [4]. Bodø is home to the second largest hospital in Northern Norway, and these students are located at that hospital [4]. This model was developed on the principle that the students followed the same schedule as that at Tromsø [3]. Academic training schedules at the two centers are largely similar, with only minor variations in learning activities. In 2017 UiT developed *the Finnmark model for medical student training*, a decentralized model wherein students complete the fifth and sixth year of medical school in the rural county of Finnmark rather than the main training site in Tromsø.

To ensure consistency in medical education, the quality of teaching must be assessed in decentralized education. Students in Bodø, Tromsø, and Finnmark undertake a common final exam. Examination results can be used to assess outcomes of students’ learnings at different training sites. Reports from UiT for the period from 2018–2023 revealed that 87.3% of the 490 students in Tromsø successfully passed the final exam. Moreover, the passing percentage of the students who received decentralized education was higher (91.2% of the 147 students in Bodø and 96% of the 50 students in Finnmark). The differences between the three learning sites are summarized in Table 1.

**Table 1:** Differences between learning sites

	**Tromsø**	**Bodø**	**Finnmark**
**Number of inhabitans**	66,281	42,831	11,310 (Hammerfest)
**Population density**	Urban	Urban	Rural
**Hospital (patient base)**	University hospital (130 000)	Regional hospital (78 000)	Local hospital (45 000)
**Study site**	Main campus	Decentralized	Decentralized
**Learning goals**	*Identical learning goals*		
**Special learning activities**		Mostly similar to Tromsø, but some more simulation and communication training	More training in NTS, cultural competency and emergency medicine. Extensive use of simulation. More of the training in general practice; both gyneocology and a practical workshop on using interpreters

The Finnmark model places an extensive focus on the acquisition of non-technical skills (NTS) via the continuous use of simulations so that the students may achieve high levels of NTS. These NTS includes situational awareness, decision-making, communication, teamwork and leadership [5]. Previous studies have demonstrated the role of NTS of the health care professionals in patient safety [6]. Insufficient NTS have been identified as a contributing factor in 70% of adverse events occurring in hospital settings [7]. NTS include interpersonal skills and complement the necessary technical skills required for clinical practice [8]. In contrast, technical skills are the profession-specific competency possessed by health professionals and students [5, 9].

Researchers have debated the use of the term NTS [10]. Nevertheless, NTS remains the most commonly used description. NTS can be acquired via training [11], and higher levels of NTS have been shown to improve patient safety [5, 12]. Therefore, health professionals and students should undergo NTS training [13].

Evaluating the outcomes of training is an essential element of high-quality training. Because providing feedback to students and health professionals on their NTS levels will aid in increasing the focus on gaining the right skills, it is necessary to develop tools to assess the NTS of health professionals and students. Tools have been used to assess NTS in the field of aviation for decades [14], and have been developed to assess the NTS of health professionals since the beginning of 2000 [8, 15]. Previously, we have developed NorMS-NTS, a tool that assesses the NTS of Norwegian medical students, in 2022 [16]. The tool is specific for the Norwegian context and evidence of validity show single measure ICC levels in the same range as other validated NTS tools [17].

The training in Finnmark emphasised training in non-technical skills using simulation to a larger extent than the two other university campuses. This study aimed to compare the NTS performance of medical students in their last year of education at three different training sites of the same university.

## Methods

### Overview

This was an observational cohort study [18]. From the three cohorts (Finnmark, Bodø and Tromsø) eight medical students from each of the training sites were studied. The experimental variable was a different training site. Eight raters who were blinded to the training site assessed the NTS performance of the 24 medical students using the NorMS-NTS tool (Table 2). We compared the results of the statistical analysis thereafter.

**Table 2:** The NorMS-NTS tool

Category^a^	Category score^b^	Element^a^	Element score^b^	Feedback
Communication		Team communication		
		Establish mutual understanding		
		Patient communication		
Situation awareness		Situational assessment		
		Understanding of team members’ roles		
		Attentiveness		
Teamwork		Professional modesty		
		Flexibility		
		Efficient use of team members		
Decision making		Uncertainty management		
		Decision analysis		
		Leadership		
		Prioritization		

General comments:

Overall global rating (Mark with a ring)

Very poor 1 – 2 – 3 – 4 – 5 – 6 – 7 Excellent

^a^*N/A *Not applicable. 1, much below average; 2, below average; 3, acceptable; 4, above average; 5, much above average

^b^Within team unless other specified

### Setting

The students at all three UiT training sites studied participate in InterSim, a simulation-based multi-professional training program that encompasses different acute care situations with standardized scenarios, during the last term of the sixth year of their undergraduate program. The medical students were paired with nursing students forming teams, which also included radiographers and bioengineering students in some cases. The team has to work together to diagnose and treat the patient in a simulated acute care setting. The medical student is the team leader. We chose to compare the students NTS when participating in InterSim, as it is a mandatory one-day course with standardized scenarios at all three campuses.

We video recorded the sessions of the medical students from Finnmark, Bodø, and Tromsø participating in two different scenarios, with each scenario lasting 12–20 min. The first scenario involved a patient with sepsis, whereas the second scenario involved a patient with postoperative dyspnea. One or two trained physicians and nurses facilitated each simulation and debriefing. Although most scenarios employed a simulated patient, some scenarios were performed using a simulation manikin owing to the COVID-19 restrictions. The students performed all measurements, examinations, and tests on the patients and gathered details regarding pain and emotions. The facilitators provided the answers subsequently. The students informed the facilitators regarding the procedures they aimed to conduct if the equipment was missing, and the facilitator provided the results.

### Video recordings

All participants were sixth-year medical students from Finnmark, Bodø, or Tromsø. When participating in the mandatory InterSim training they were asked if they wanted to participate in the project. We provided a thorough explanation regarding the objectives and aims of the study to the participants. Participation was voluntary and had no consequences for their education. We randomly selected eight video recordings with sufficient sound and image quality from the video recordings of over 100 teams acquired between 2018 and 2023. We designated identification numbers to the videos and rfnational simulation networks (InterRegSim, a national collaboration for simulation-based learning in the specialist health service in andomized their order. The teaching and training did not vary throughout the years, but there was different emphasis on training non-technical skills and use of simulation between study sites.

### Raters

We contacted two national simulation networks (InterRegSim, a national collaboration for simulation-based learning in the specialist health service in Norway [19] and the Better & Systematic Team training [BEST] network, an international multi-professional team training program originating in Finnmark [20]) via email to recruit raters. These were chosen because they work with team training and simulation and are not part of the program. We offered gift cards of NOK 3000 to the 11 raters recruited. We recruited more raters than necessary to compensate for dropouts. Eight raters, comprising four men and four women aged 46–69 years (mean: 55 years), completed the task within a specified timeframe.

The raters had 22–44 (mean: 27.5) years of clinical experience; two raters did not answer this question. Six raters were registered nurses, whereas two were medical doctors. Seven raters reported prior experience with the NTS and/or NTS tools; the last rater did not answer this question.

All raters familiarized themselves with the NorMS-NTS tool and received a presentation of the tool from a researcher (KP) via Microsoft Teams. All raters received secure online access to the 24 videos. We assigned a two-digit study identification code to each video and a number to each rater. The raters who were blinded to the training sites studied rated all videos using the NorMS-NTS tool and returned the ratings to a researcher (KP) via e-mail. The raters were only aware of the identification numbers of the students.

### Sample size

The NorMS-NTS is a new tool, and no previous study has assessed the NTS of Norwegian medical students. Consequently, we could not obtain any estimates of the prevalence or standard deviation or calculate the sample size [21]. The practical implications of the selected sample size were also important. Three raters rated 20 videos of the same length as those used in the present study in a previous study [17] and exhibited a nearly identical threshold for the number of videos each rater could rate. The assessment process is time-consuming and requires a focused rater. Therefore, we selected 24 videos and increased the number of raters to eight to increase the likelihood of accurately measuring the NTS performance levels [22].

### The NorMS-NTS tool

The NorMS-NTS tool used in this study was developed to assess the NTS of Norwegian medical students via observation in 2022 (Table 2) [16]. This tool consists of four categories comprising 13 elements rated on a 5-point Likert scale. The global overall score is rated on a 7-point Likert scale.

### Statistical analysis

We analyzed all data extracted from the NorMS-NTS forms of the raters using Statistical Analysis System (SAS 9.4). We compared the mean element-level, category-level, and overall NTS performance levels at different training sites subsequently. The null hypothesis was that no significant differences would be observed among the NTS performance of the students in the three cohorts. Tukey’s test [23] which is a test that adjust for type I-error was used to calculate significant difference [24].

## Results

### Overall NTS performance

**Fig. 1:**
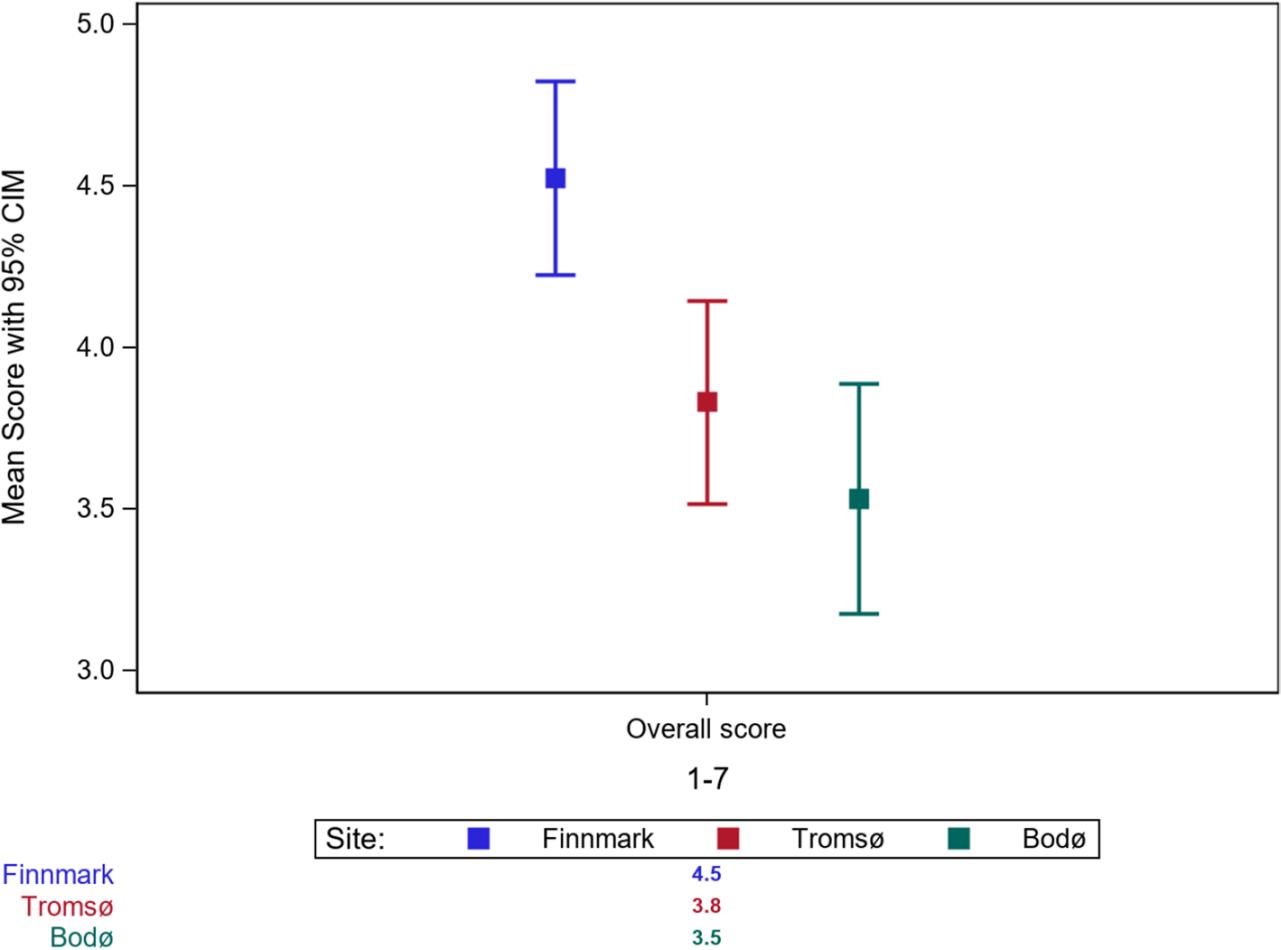
Overall score of NTS performance between the three training sites (CIM: Confidence interval of the mean)

The NTS performance levels of the medical students from Finnmark (mean 4.52 (0.25)) were significantly higher than those of the students from Bodø (mean 3.53 (0.25)) and Tromsø (mean 3.83 (0.25)) on a scale ranging from 1 to 7 (Table 3 and Fig. 1). However, we observed no significant difference between the NTS performance levels of the medical students from Tromsø and Bodø.

**Table 3:** Observed differences in NTS performance between the three training sites. Significant when *p* < 0.05

Category/element	Finnmark	Tromsø	Bodø	Finnmark-Bodø		Finnmark-Tromsø		Tromsø-Bodø	
	Mean (SD)	Mean (SD)	Mean (SD)	Mean diff. (95% CI)	*P*-value	Mean diff. (95% CI)	*P*-value	Mean diff. (95% CI)	*P*-value
Overall score	4.52 (0.25)	3.83 (0.25)	3.53 (0.25)	0.99 (0.51—1.47)	< .0001	0.69 (0.21—1.17)	0.0023	0.30 (-0.18—0.78)	0.31
**Category**									
Communication	3.59 (0.15)	3.01 (0.15)	2.96 (0.15)	0.63 (0.31—0.96)	< .0001	0.58 (0.26—0.90)	0.0001	0.05 (-0.27—0.38)	0.92
Situational assessment	3.58 (0.15)	3.26 (0.15)	3.04 (0.15)	0.54 (0.23—0.85)	0.0002	0.33 (0.02—0.63)	0.04	0.21 (-0.10—0.52)	0.24
Team work	3.73 (0.11)	3.12 (0.14)	2.96 (0.14)	0.78 (0.50—1.06)	< .0001	0.62 (0.33—0.90)	< .0001	0.16 (-0.12—0.44)	0.35
Decision Making	3.59 (0.16)	3.13 (0.15)	2.72 (0.15)	0.87 (0.52—1.21)	< .0001	0.46 (0.11—0.80)	0.0056	0.41 (0.06—0.76)	0.02
**Element**									
Understanding of team members role	3.15 (0.14)	2.84 (0.16)	2.84 (0.16)	0.30 (-0.04—0.62)	0.10	0.31 (-0.04—0.65)	0.09	0.00 (-0.35—0.34)	1.00
Attentiveness	3.53 (0.14)	3.31 (0.15)	3.15 (0.15)	0.38 (0.07—0.70)	0.01	0.22 (-0.10—0.53)	0.24	0.17 (-0.15—0.48)	0.43
Professional modesty	3.60 (0.10)	3.14 (0.12)	3.16 (0.12)	0.44 (0.16—0.72)	0.0008	0.46 (0.18—0.75)	0.0004	-0.02 (-0.30—0.26)	0.98
Flexibility	3.48 (0.13)	3.01 (0.15)	2.80 (0.15)	0.68 (0.36—0.99)	< .0001	0.47 (0.15—0.78)	0.0016	0.21 (-0.11—0.53)	0.26
Efficient use of team members	3.36 (0.13)	3.10 (0.16)	3.13 (0.16)	0.23 (-0.11—0.57)	0.24	0.26 (-0.08—0.60)	0.17	-0.03 (-0.37—0.31)	0.98
Uncertainty management	3.45 (0.19)	3.08 (0.18)	2.81 (0.18)	0.64 (0.28—1.00)	0.0001	0.37 (0.01—0.73)	0.04	0.27 (-0.09—0.63)	0.19
Decision Analysis	3.37 (0.14)	2.94 (0.16)	2.69 (0.16)	0.68 (0.34—1.02)	< .0001	0.43 (0.09—0.77)	0.009	0.25 (-0.09—0.59)	0.20
Leadership	3.34 (0.19)	3.03 (0.22)	2.70 (0.22)	0.64 (0.27—1.02)	0.0002	0.31 (-0.06—0.69)	0.12	0.33 (-0.04—0.71)	0.10
Prioritization	3.29 (0.20)	3.12 (0.19)	2.80 (0.19)	0.49 (0.10—0.87)	0.0098	0.17 (-0.22—0.56)	0.56	0.32 (-0.07—0.71)	0.13

*SD* Standard deviation, *Diff.* Difference, *CI* Confidence interval

### NTS performance at the category level

**Fig. 2:**
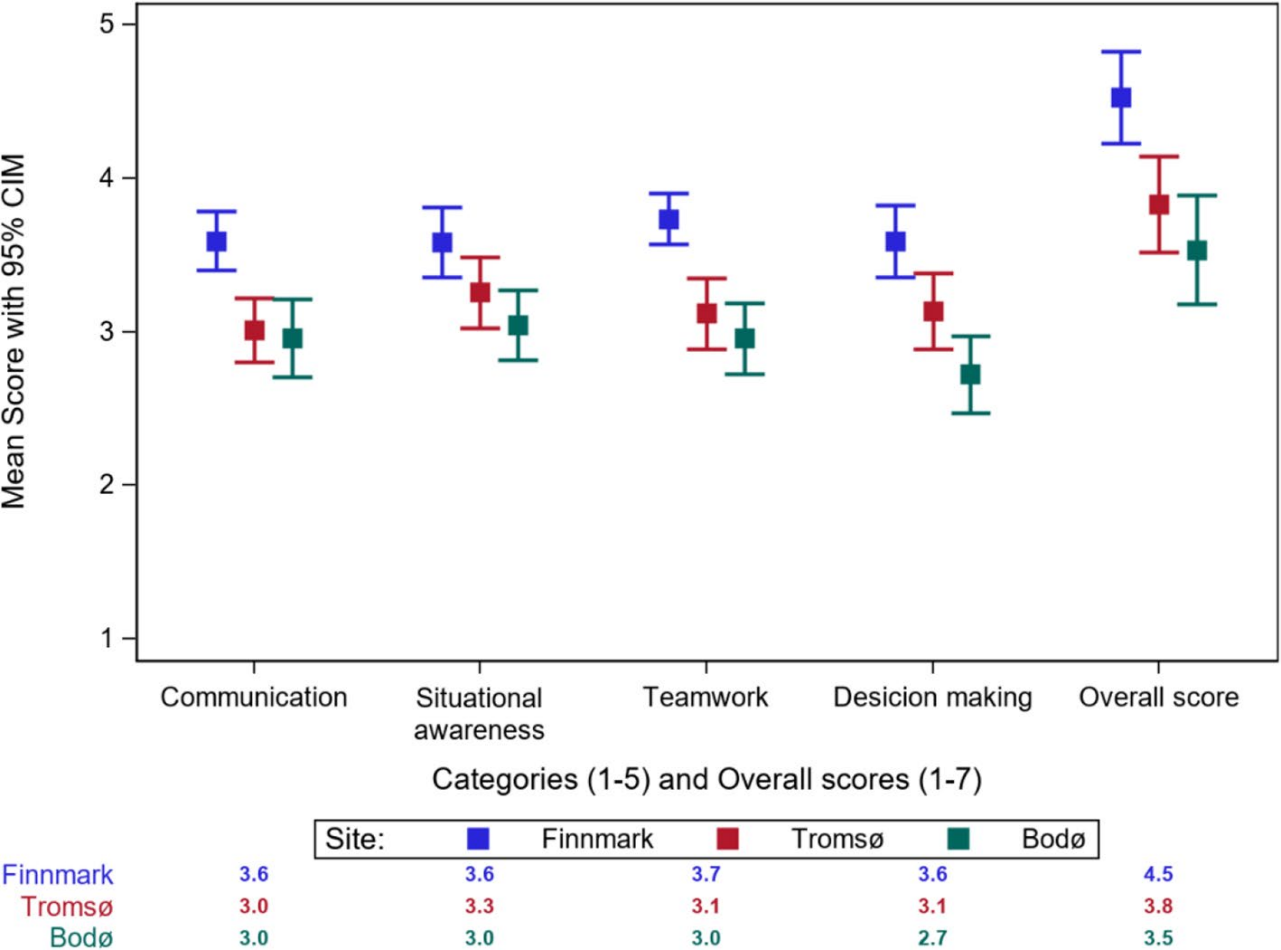
Category score of NTS performance between the three training sites (CIM: Confidence interval of the mean)

The NTS performance levels of the students from Finnmark were significantly higher than those of the students from Bodø and Tromsø in all categories (Fig. 2). We observed no significant difference between the NTS performance levels of the students from Bodø and Tromsø, except in terms of the category of “decision making”. The NTS performance levels of the students from Bodø were significantly lower than those of the students from Tromsø for this category. The categories of “Communication” and “Teamwork” exhibited the most significant differences between Finnmark and the other training sites. These results were also correlated with the overall score.

### NTS performance when comparing elements

**Fig. 3:**
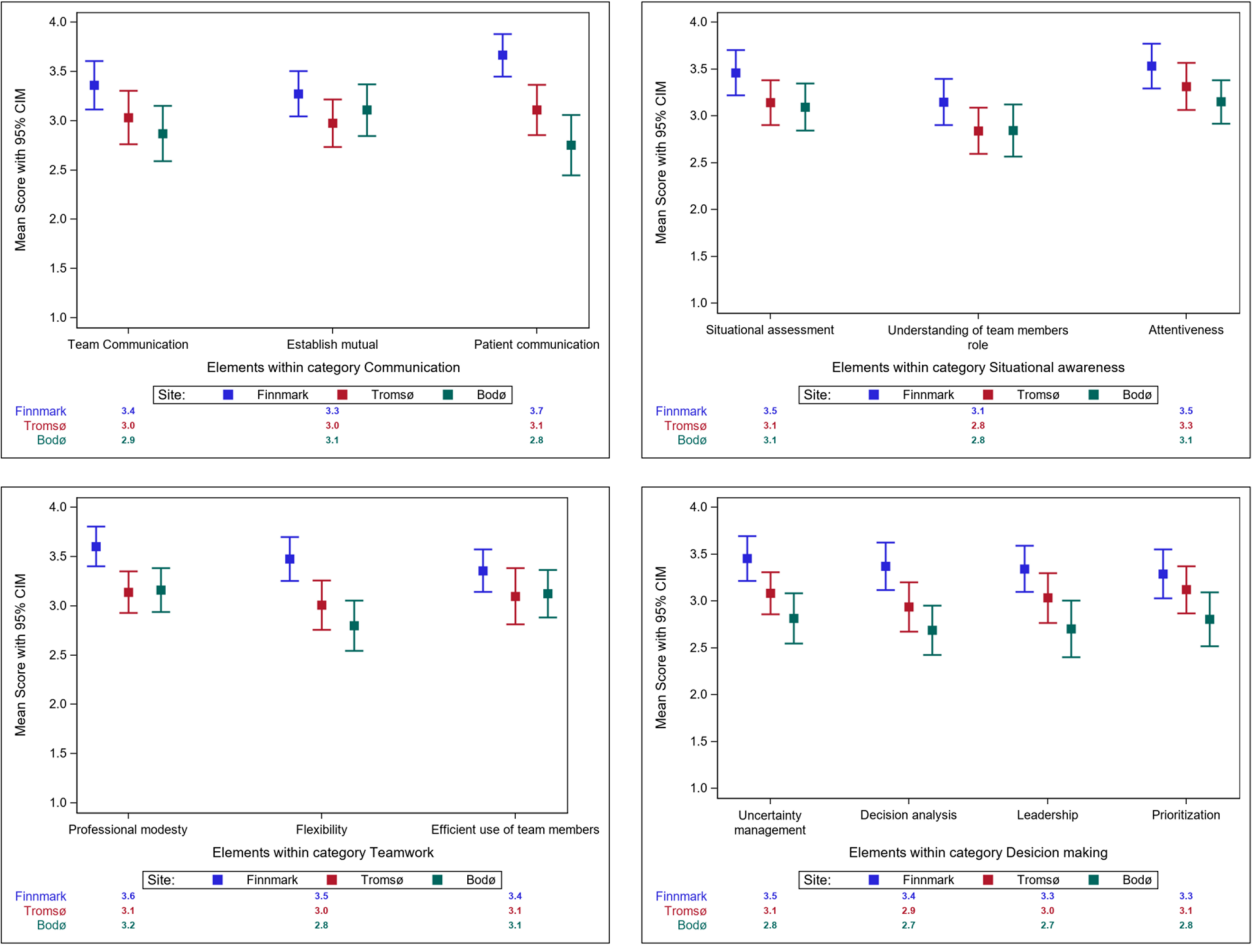
Element score of NTS performance between the three training sites (CIM: Confidence interval of the mean)

The NTS performance levels of the students from Finnmark were significantly higher than those of their peers in Bodø in all elements (Table 3), except the following elements: “Establish mutual understanding,” “Understanding of team members role” and “Efficient use of team members” (Fig. 3). The element “Patient communication” exhibited the most significant difference. The scores for “Patient communication,” “Professional modesty,” “Flexibility,” “Uncertainty management,” and “Decision analysis” of the students from Finnmark were significantly higher than those of the students from Tromsø. We observed no significant difference between the students from Tromsø and Bodø in terms of these elements.

## Discussion

The present study demonstrated that the overall and category-level NTS performance levels of the students from Finnmark were significantly higher than those of the students from Bodø and Tromsø. Moreover, we observed no significant difference between the NTS performance levels of the students from Tromsø and Bodø, except in the category “Decision making.” The scores of the students from Bodø for this category were significantly lower than those of the students from Tromsø. The NTS performance level of the students from Finnmark was significantly higher for the elements “Patient communication,” “Professional modesty,” “Flexibility,” “Uncertainty management,” and “Decision analysis.” This finding of consistent results, with no differences between the NTS performance of the students from Bodø and Tromsø, and significantly better NTS performance of the students from Finnmark supports the internal consistency of the tool. The present study is novel in that no previous study has evaluated the NTS performance of Norwegian medical students receiving decentralized medical education. Hence, the results of previous studies cannot be compared with our results.

UiT aimed to use multi-professional team training to enhance the NTS when developing the Finnmark model. The findings of the present study, a follow-up study conducted to evaluate its effect, indicate that the NTS performance levels of the students from Finnmark were superior. However, this finding can be attributed to several reasons.

First, selection bias may have influenced our results. All students in Finnmark and Bodø actively elected to pursue decentralized medical education during the fifth and sixth years of study. The students electing to go to rural areas may possess some distinct features prior to their choice that are associated with higher NTS levels. The student groups in Finnmark are smaller than those in Bodø and Tromsø. Small group teaching optimizes learning in healthcare [25]. The knowledge of the students increases when they can build their understanding with their peers [25]. Small group teaching also promotes team-building skills [25]. The student group in Finnmark is smaller; consequently, the lectures also contained small groups of students. Small-group teaching and small-group lectures are not equivalent [25]. However, the learning experience is correlated with the engagement of the students. Thus, small group lectures may facilitate higher learning outcomes than the bigger groups in Tromsø and Bodø. Furthermore, smaller groups may facilitate active participation, “face-to-face” contact between participants, and purposeful activities, which are the three key elements for small group teaching [25].

More of the learning occurs in general practice in Finnmark, with general practitioners acting as teachers. The students received one-on-one half-day training in gynecology from an experienced general practitioner. The students also participated in general practice group workshops. Three students and one experienced general practitioner consulted with patients requiring a medical interpreter. The patient had regular appointments, and one of the students regularly consulted with a medical interpreter via telephone. The remaining students and general practitioners observed the consultation and participated in discussions. All students conducted one consultation by themselves. These new teaching models may have affected NTS positively as students are more engaged and active in the process and will receive feedback on different level that might help them with NTS. Those effects warrant further studies.

Another noteworthy difference is that the training site located in Finnmark is a small local hospital, whereas the training sites located in Tromsø and Bodø are a large University hospital and a large regional hospital, respectively. The local hospital in Finnmark comprised more generalists, whereas regional and university hospitals comprised more branch specialists. Consequently, more generalists trained the students in Finnmark, which may have affected the results. Students are expected to be skilled professional generalists by the time they graduate from medical school with the ability to become lifelong learners. The outcomes may be affected if education is particularly narrow or specialized. There is also a possibility that the teachers and the whole community in rural Finnmark places higher value on NTS. Further studies should aim to clarify these findings.

### Limitations

The scenarios were standardized. However, they were not performed in an identical manner by the facilitators as they had different levels of training and different ways of performing their roles. Facilitators may have affected the ability of the students to perform at their highest level negatively and positively. Notably, several facilitators were involved at each institution to mitigate the influence of individual facilitators.

This study included eight students randomly selected from each training site. Ideally, all students should have been assessed; however, this was not possible owing to practical limitations. We obtained 64 scores for each element, category, and the global score of the NorMS-NTS at each training site as eight raters participated in this study. The wide range of years the students were video recorded may also have influenced the outcome. The teaching and training did not vary throughout the years, but there was different emphasis on training non-technical skills and use of simulation between study sites. However, the generalizability of our findings remains unknown. Further studies must be conducted to validate these results and assess their applicability.

The NorMS-NTS is a novel assessment tool used to evaluate the NTS of Norwegian medical students. The process of collecting evidence for its validity is ongoing. Although not proven optimal for summative assessment, it is the only tool available to assess the NTS of Norwegian medical students. This may have affected the results. However, with one exception, we found no statistical difference between the NTS performance levels of the students from Bodø and Tromsø, which supports the reliability of the tool.

## Conclusion

The NTS performance levels of the medical students in Finnmark were significantly higher than that of the students in Bodø and Tromsø. Further studies must explore the reasons for this discrepancy. However, our study demonstrated that rural decentralized medical education may yield better learning outcomes than standard education in large, centralized hospitals.

### Supplementary Information

Supplementary Material 1.

## Data Availability

Data is provided within the manuscript  and supplementary files.
